# Single‐frequency ultrasonic extraction of bioactive chlorogenic acid from heilong48 soybean variety: Parametric optimization and comprehensive evaluation of physicochemical and bioactive properties

**DOI:** 10.1002/fsn3.2593

**Published:** 2021-10-31

**Authors:** Nelson Dzidzorgbe Kwaku Akpabli‐Tsigbe, Yongkun Ma, John‐Nelson Ekumah, Juliet Osabutey, Jie Hu, Manqing Xu, Nana Adwoa Nkuma Johnson

**Affiliations:** ^1^ School of Food and Biological Engineering, Oversea College of Education Jiangsu University Zhenjiang China; ^2^ Department of Nutrition and Food Science, College of Basic and Applied Sciences University of Ghana Legon Ghana; ^3^ Department of Early Childhood Education University of Education Winneba Ghana; ^4^ Virtuous Experimental School Achimota‐Accra Ghana

**Keywords:** chlorogenic acid, heilong48 soybean variety, optimization, physicochemical properties, ultrasonic‐assisted extraction

## Abstract

Chlorogenic acid (CA), especially that found in soybean, is a rich bioactive compound but has received very little attention in research settings in past decades. Ultrasonic‐assisted extraction (UAE) could be an efficient method to increase CA release from soybean. Hence, this study aimed to optimize UAE parameters for CA extraction from heilong48 soybean (HS) variety with comprehensive physicochemical and bioactive properties evaluation. Optimization of ultrasound parameters with Box‐Behnken design (BBD) found frequency (20.0 kHz), power density (30.0 W/L), temperature (37.9℃), and time (28.0 min) as the significant optimized parameters, which gave CA yield of 5.007 ± 0.033 mg/g and DPPH of 93.197 ± 0.213 μmol AA eq/g dry sample and were higher than that of nonultrasound‐treated (raw) HS sample (CA yield, 1.627 ± 0.528 mg/g, and DPPH, 10.760 ± 0.207 μmol AA eq/g dry sample). A satisfactory model was obtained. SEM results confirmed the structural alterations of HS variety caused by the optimized UAE parameters. High TPC (4.726 ± 0.002 mg GAE/g), TPA (1.883 ± 0.005 mg GAE/g), and low TFC (0.040 ± 0.008 mg RE/g) were obtained. A positive linear correlation between antioxidant activity and TPC was established. Protein–phenolic interaction in HS variety was observed. The results proposed that polyphenols should be considered as a significant component of HS variety. Likewise, HS variety could be utilized for CA extraction.

## Graphical abstract

A satisfactory ultrasonic‐assisted extraction model was obtained. Heilong48 soybean variety could be used for CA extraction. Protein–polyphenol interaction was observed in the heilong48 soybean variety.

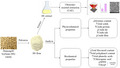

## 1 INTRODUCTION

Soybean (*Glycine max* L.), from the family of Fabaceae, is a good, rich, and vital dietary source of protein, lipids, minerals, vitamins, fiber, and bioactive compounds. Its protein too is rich in essential amino acids (Messina & Messina, 2010). Due to its exceptional nutritional value, soybean is a vital staple crop in the world. Globally, the role of legumes in traditional diets of various regions is significant. Messina and Messina (2010) have shown that soybean is the only leguminous crop, containing in high quantities substances (such as alpha‐linolenic acid; Blondeau et al., 2015) that are beneficial to humans, with regards to preserving the heart and/or blood vessel. Furthermore, polyphenolic compounds in significant quantities are found in soybeans, making them unique among legume crops (Sakthivelu et al., 2008). Kim et al. (2012) found soybeans to contain isoflavones and phenolic acids (tocopherols, saponins, and phytic acids). Phenolic acids have lately gained considerable attention, in research settings, due to their practicable biological effects. Chlorogenic acid (CA) is one most significantly available phenolic acids found in soybean. CA is an essential bioactive dietary polyphenol that plays important therapeutic roles including antibacterial, antioxidant activity, anti‐inflammatory, cardioprotective, neuroprotective, antiviral, anti‐hypertension, diabetes, and anti‐obesity (Liang & Kitts, 2016; Santana‐Gálvez et al., 2017).

Despite the health/therapeutic advantages of CA, existing literature shows a dearth of information on CA obtained from soybean, as the majority of available data concentrate on the metabolism and bioactivity of other bioactive compounds found in soybean (Cao et al., 2019; Křížová et al., 2019). Thus, the majority of these studies dwelled much on substances like isoflavones from soybean meal (Wang et al., 2009), with neglect for other bioactive (phenolic) compounds such as CA. This is justified with isoflavones mentioned in titles or abstracts of over 17,000 academic articles in Web of Knowledge (Chen et al., 2012) with no mention of CA, clearly supporting the lack of information (knowledge gap) in literature. Recently, a new soybean variety (heilong48) was introduced with improved qualities by the Tianxia Agricultural and Sideline Products in China. This variety was developed to have a high phenolic acids value over other soybean varieties. The heilong48 soybean (HS) variety has not been investigated for CA extraction, and its constituents may vary regarding other varieties, from a producer to another due to differences in genetic makeup, soil, and environmental conditions. The CA content of HS variety can be extracted with ultrasound for enhanced yield and food/pharmaceutical applications.

Extraction of numerous plant metabolites kinds worthful as ingredients for food, medicinal, and beautifying industries could be done with ultrasound‐assisted extraction (UAE). Alves Filho et al. (2020) studied ultrasound utilization for metabolites extraction from numerous vegetables, fruits, stems, leaves, and agricultural waste, for example, blackberries, acerola, olive leaves, sweet potatoes, jabuticaba, potato peel, among others. UAE is simple, nonthermal, fast, and low cost hence applicable for rapid extraction of numerous phenolic compounds from plant matrix with no decay into other inactive compounds. However, some researchers have reported on the degradation of anthocyanins by overexposure to the waves and cavitation created by ultrasound (Alves Filho et al., 2020). As a result, UAE application needs optimization for each material type to be processed; else, phenolic compounds may undergo different degradation degrees or sonochemical metamorphosis. Correct selection of UAE parameters makes it a useful method not only as a method for extraction but selective extraction of different compounds too, from herbs, vegetables, and fruits, for example, purple sweet potato, potato peels, and microalgae (Alves Filho et al., 2020). The present study, therefore, aimed at optimizing ultrasonic parameters for UAE of CA from HS variety and evaluating the physicochemical and bioactive characteristics of the soybean variety.

## 2 MATERIALS AND METHODS

Tianxia Agricultural and Sideline Products, China, supplied the HS variety used in the study. Only analytical grade chemicals bought from Sinopharm Chemical Reagent Co., Limited (China) were utilized in this study.

### 2.1 Preparation of flour from HS variety

Heilong48 soybean flour was prepared according to the method outlined in our previous works (Akpabli‐Tsigbe, Ma, Ekumah, Osabutey, Hu, Xu, & Johnson, 2021; Akpabli‐Tsigbe, Ma, Ekumah, Osabutey, Hu, Xu, Johnson, et al., 2021), packed in weights of 150 g in zip‐lock rubber bags, and stored (−20℃) for further studies.

### 2.2 HS extract preparation

The HS extract was prepared following the method outlined by Adane et al. (2019) and used for the determination of total polyphenol contents (TPC), total flavonoids contents (TFC), total phenolic acids (TPA), and antioxidant activity.

### 2.3 UAE of CA

The UAE of CA from the HS variety was performed at three levels of frequency (20, 40 and 60 kHz) of ultrasonic water bath (Miebo Biotech. Co.) operated at different power levels (180, 240 and 300 W) of maximum effective ultrasonic power density (30, 40 and 50 W/L respectively) at different temperature levels (35, 40, and 45℃), for different extraction time levels (25, 30, and 35 min) (Table S1a). 40 mg of HS flour was accurately weighed into glass tubes and hydrated with 30 ml of distilled water resulting in a solution of 1.33 mg/ml. Each sample tube was treated with ultrasound in an ultrasonic water bath, positioned above the ultrasonic transducer of the bath to ensure that ultrasonic intensity for each experimental run was the same. The extracts obtained after sonication were centrifuged for 20 min at 4000 *g* with RJ‐TDL‐50A centrifuge manufactured by Ruijiang Analytical Instrument Corporation Limited (China), filtered, and stored at −20℃ for additional works.

### 2.4 Optimization of ultrasonic parameters for CA extraction using Box‐Behnken design

Optimization was performed to obtain maximum yield of CA with improved antioxidant activity using a desirability index. A Box‐Behnken design (BBD) using response surface methodology (RSM) was applied to model and optimize sonication parameters and their influences on CA yield and antioxidant activity as responses. The independent parameters used were frequency (A), power density (B), temperature (C), and time (D), varying from 20 to 60 kHz, 30 to 50 W/L, 35 to 45℃, and 25 to 35 min respectively. Selection of the levels of the parameters was based on literature (Alves Filho et al., 2020; Esclapez et al., 2011; Guglielmetti et al., 2017). BBD with four‐factor‐three‐level was used, which gave 29 experimental runs performed on HS extract. Correlation of the association of each independent variable to the dependent variable was achieved through the fitting of a second‐degree polynomial model from the equation:

(1) $$Y=\beta_{0}+\sum_{i=1}^{3}\beta_{i}X_{i}+\sum_{i=1}^{3}\beta_{\mathrm{ii}}{X_{i}}^{2}+\sum_{i=1}^{3}\times \sum_{j=i+1}^{3}\beta_{\mathrm{ij}}X_{i}X_{j}\;\;\;\;\;\;\;\;\;\;\;\;\;\;\;\;\;\;\;\;\;\;\;\;\;\;\;\;\;\;\;\;\;\;\;\;\;\;\;\;\;\;\;\;\;\;$$

where, *Y* = predicted dependent parameter, *β*_0_ = intercepts, *β_i_* = model's linear regression coefficients, *β_ii_* = model's second‐order regression coefficients, and *β_ij_* = model's estimated interaction regression coefficients. *X_i_* and *X_j_* = values of factors. The equation below was used for the selection of the optimized parameters based on the overall desirability index (DI):

(2) $$DI={\left[\prod_{i=1}^{3}d_{i}\left(y_{i}\right)\right]}^{1\;/\;3}\;\;\;\;\;\;\;\;\;\;\;\;\;\;\;\;\;\;\;\;\;\;\;\;\;\;\;\;\;\;\;\;\;\;\;\;\;\;\;\;\;\;\;\;\;\;\;\;\;\;\;\;\;\;\;\;\;\;\;\;\;\;\;\;\;\;\;\;\;\;\;\;\;\;\;\;\;\;\;\;\;\;\;\;\;\;\;\;\;\;\;\;\;\;\;\;\;\;\;\;\;\;\;\;\;\;\;\;\;\;\;\;\;\;\;\;\;\;\;\;\;\;\;\;\;$$

where, *d_i_* = dependent variable's desirability index (0–1), *y_i_* = dependent variables.

### 2.5 Standard CA solution preparation

The standard CA solution was prepared using the protocol reported by Akpabli‐Tsigbe, Ma, Ekumah, Osabutey, Hu, Xu, Johnson, et al. (2021) and validated against Beer‐Lambert's law.

### 2.6 Proximate analysis

Moisture content, total solids, fat, protein, crude fiber, and ash were determined with the methods delineated by AOAC (2005).

### 2.7 TPC, TFC, TPA, and antioxidant determination

Total polyphenol contents and TFC were determined using the technique reported by Kwaw et al. (2017). The antioxidative activity of the HS variety was determined by 2,2‐diphenyl‐1‐picrylhydrazyl (DPPH) following the protocol outlined by Haida and Hakiman (2019) and ferric reducing antioxidant power capacity (FRAP) following the procedure reported by Chaves et al. (2020). TPA was determined by adopting the method described by Haida and Hakiman (2019).

### 2.8 CA determination

Chlorogenic acid determination was performed according to the method of Adane et al. (2019). CA concentration was computed against the stock solution utilizing Beer‐Lambert's Law at *λ*_max_ = 325 nm (maximum wavelength). CA content and %CA in samples were calculated using Equations (3) and (4) respectively.

(3) $$\mathrm{CA}\;\mathrm{content}\;\left(\mathrm{mg}\right)=\frac{\left[\mathrm{CA}\;\mathrm{conc}\;\left(\mathrm{mg}/L\right)\right]\times {\left[\mathrm{total}\;\mathrm{sample}\;\mathrm{volume}\;\left(\mathrm{ml}\right)\right]}^{2}}{\left[\mathrm{measured}\;\mathrm{sample}\;\mathrm{volume}\;\left(\mathrm{ml}\right)\right]\times 1000}\;\;\;\;\;\;\;\;\;\;\;\;\;\;\;\;\;\;\;\;\;\;\;\;\;\;\;\;\;\;\;\;\;\;\;\;\;$$

(4) $$\%\mathrm{CA}\;\left(w/w\%\right)\;=\frac{\left[\mathrm{calculated}\;\mathrm{mass}\;\mathrm{of}\;\mathrm{CA}\;\left(\mathrm{mg}\right)\right]}{\left[\mathrm{mass}\;\mathrm{of}\;\mathrm{sample}\;\mathrm{measured}\;\left(\mathrm{mg}\right)\right]}\times 100\%\;\;\;\;\;\;\;\;\;\;\;\;\;\;\;\;\;\;\;\;\;\;\;\;\;\;\;\;\;\;\;\;\;\;\;\;\;\;\;\;\;\;\;\;\;\;\;\;\;\;\;\;$$

### 2.9 Statistical analysis

Design Expert (STAT‐EASE Inc.) software version 11.0.5.0 was utilized for both the experimental designs and statistical analysis (optimization). P test, determination coefficient (*R* ^2^), variation coefficient (CV), and lack of fit test at *p* < .05, .01, and .001 were used to assess the accuracy of the model. Data were reported as mean ± standard deviations of three independent determinations. V2018 OriginPro (OriginLab) was used to construct graphs and MINITAB (Minitab Inc.) version 18.1 program to compute analysis of variance (ANOVA) with Tukey's test applied at *p* < .05 to compare the averages. Correlations between parameters were computed with Pearson's correlation. Parameters’ relationships were computed with the principal component analysis (PCA).

## 3 RESULTS AND DISCUSSION

The UAE was done to investigate the probabilities of extracting bioactive CA with high yield from HS variety. Four ultrasonic factors were experimented with at three levels using BBD to obtain higher yield of CA with enhanced DPPH from the HS sample (Table 1). A quadratic polynomial model was fitted to the dependent parameters, and ANOVA was applied to evaluate the factors’ effects, interactions, and model significance statistically. Table 2 showed the model *p*‐ and *F*‐values of the regression coefficients of the response parameters. Generally, factors exhibiting high significant effects have greater *F*‐values and smaller *p*‐values (Zhou et al., 2015). The large *F*‐values signified a significant model and that only 0.01% chance could occur by noise. Similarly, the high *R* ^2^ values of .9967 (for CA yield) and .9239 (for DPPH) signified a significant model. It also indicated that 92.39% and 99.67% of the total variations for the DPPH and CA yield respectively were due to the independent parameters. Furthermore, the lack of fit values of the models was insignificant statistically. The quadratic polynomial model, therefore, was a good estimations (model fitness) of the responses and explained the responses sufficiently.

**TABLE 1:** Box‐Behnken design (BBD) matrix with experimental design and data for ultrasonic‐assisted extraction of chlorogenic acid from heilong48 soybean (HS) variety

Run	Ultrasonic parameters (actual and coded values)				Chlorogenic acid (CA) yield (mg/g)		DPPH (μmol AA eq/g dry sample)	
	Frequency (kHz)	Power density (W/L)	Temperature (^o^C)	Time (min)	Experimental values	Predicted values	Experimental values	Predicted values
	A	B	C	D	CA_(Actual)_	CA_(Predicted)_	DPPH_(Actual)_	DPPH_(Predicted)_
1	60 (+1)	40 (0)	40 (0)	35 (+1)	4.09 ± 0.01	4.03	90.93 ± 0.07	91.10
2	40 (0)	40 (0)	40 (0)	30 (0)	2.52 ± 0.01	2.44	92.92 ± 0.09	92.82
3	40 (0)	40 (0)	45 (+1)	25 (−1)	3.06 ± 0.02	3.07	91.11 ± 0.03	91.17
4	40 (0)	30 (−1)	35 (−1)	30 (0)	3.97 ± 0.01	3.92	91.50 ± 0.16	91.52
5	40 (0)	50 (+1)	40 (0)	35 (+1)	4.79 ± 0.02	4.81	91.07 ± 0.03	91.26
6	60 (+1)	40 (0)	40 (0)	25 (−1)	4.01 ± 0.02	4.02	91.79 ± 0.21	92.18
7	20 (−1)	40 (0)	40 (0)	35 (+1)	3.15 ± 0.01	3.01	93.76 ± 0.03	93.42
8	60 (+1)	50 (+1)	40 (0)	30 (0)	4.43 ± 0.01	4.44	91.66 ± 0.06	91.63
9	60 (+1)	40 (0)	45 (+1)	30 (0)	4.85 ± 0.01	4.89	92.13 ± 0.09	92.07
10	40 (0)	50 (+1)	35 (−1)	30 (0)	3.52 ± 0.00	3.48	91.00 ± 0.03	91.02
11	40 (0)	40 (0)	35 (−1)	35 (+1)	3.19 ± 0.01	3.22	90.01 ± 0.12	90.02
12	20 (−1)	40 (0)	45 (+1)	30 (0)	2.49 ± 0.01	2.55	92.36 ± 0.06	92.60
13	40 (0)	40 (0)	40 (0)	30 (0)	2.42 ± 0.02	2.44	92.05 ± 0.03	92.82
14	20 (−1)	40 (0)	35 (−1)	30 (0)	4.44 ± 0.02	4.50	92.41 ± 0.12	92.36
15	40 (0)	40 (0)	35 (−1)	25 (−1)	2.99 ± 0.03	2.97	91.38 ± 0.10	91.75
16	60 (+1)	30 (−1)	40 (0)	30 (0)	4.86 ± 0.02	4.83	92.54 ± 0.09	92.43
17	40 (0)	40 (0)	45 (+1)	35 (+1)	2.30 ± 0.01	2.35	93.00 ± 0.06	92.69
18	20 (−1)	50 (+1)	40 (0)	30 (0)	3.97 ± 0.01	4.04	92.76 ± 0.09	92.94
19	40 (0)	30 (−1)	40 (0)	35 (+1)	1.34 ± 0.00	1.43	92.95 ± 0.09	93.22
20	20 (−1)	40 (0)	40 (0)	25 (−1)	3.56 ± 0.02	3.49	92.66 ± 0.09	92.54
21	40 (0)	30 (−1)	40 (0)	25 (−1)	4.99 ± 0.02	5.06	92.51 ± 0.07	92.20
22	20 (−1)	30 (−1)	40 (0)	30 (0)	3.66 ± 0.01	3.68	93.70 ± 0.03	93.80
23	40 (0)	40 (0)	40 (0)	30 (0)	2.39 ± 0.01	2.44	93.09 ± 0.12	92.82
24	60 (+1)	40 (0)	35 (−1)	30 (0)	3.68 ± 0.02	3.71	90.57 ± 0.10	90.21
25	40 (0)	40 (0)	40 (0)	30 (0)	2.48 ± 0.02	2.44	92.99 ± 0.14	92.82
26	40 (0)	40 (0)	40 (0)	30 (0)	2.40 ± 0.01	2.44	93.03 ± 0.06	92.82
27	40 (0)	50 (+1)	45 (+1)	30 (0)	3.59 ± 0.02	3.52	91.70 ± 0.12	91.74
28	40 (0)	30 (−1)	45 (+1)	30 (0)	3.20 ± 0.03	3.11	92.86 ± 0.07	92.90
29	40 (0)	50 (+1)	40 (0)	25 (−1)	1.64 ± 0.02	1.65	92.89 ± 0.06	92.50

**TABLE 2:** ANOVA, regression analysis and optimal conditions for ultrasonic‐assisted extraction of CA from HS variety

Source	CA yield (mg/g)		DPPH (μmol AA eq/g dry sample)	
	*F*‐value	*p*‐value	*F*‐value	*p*‐value
Model	303.73	<.0001***	12.13	<.0001***
Linear				
A: Frequency	285.19	<.0001***	39.38	<.0001***
B: Power density	0.0844	.7757^NS^	15.15	.0016*
C: Temperature	69.77	<.0001***	24.16	.0002**
D: Time	25.48	.0002**	0.2347	.6355^NS^
Interactions				
AB	21.67	.0004**	0.0066	.9364^NS^
AC	385.17	<.0001***	4.75	.0469*
AD	9.50	.0081*	7.04	.0189*
BC	27.92	.0001***	0.7980	.3868^NS^
BD	1829.63	<.0001***	9.36	.0085*
CD	36.47	<.0001***	19.47	.0006**
Quadratic				
A^2^	1,251.28	<.0001***	0.1253	.7287^NS^
B^2^	503.41	<.0001***	0.2171	.6484^NS^
C^2^	136.96	<.0001***	43.47	<.0001***
D^2^	9.07	.0093*	9.74	.0075*
Fitting statistics				
Lack of fit	2.44	.2028^NS^	0.6206	.7539^NS^
*R* ^2^	.9967		.9239	
Adjusted *R* ^2^	0.9934		0.8477	
Predicted *R* ^2^	0.9830		0.6867	
Adeq. precision	63.5276		14.2280	
C.V. %	2.35		0.4007	
Mean	3.38		92.18	
Standard dev.	0.0795		0.3694	
Optimization equations				
$\mathrm{CA}\;\mathrm{yield}\;\left(\mathrm{mg}/g\right)=2.44+0.3875A‐0.1917C‐0.1158D‐0.185\mathrm{AB}+0.78\mathrm{AC}+0.1225\mathrm{AD}+0.21\mathrm{BC}+1.7\mathrm{BD}‐0.24\mathrm{CD}+1.1A^{2}+0.7003B^{2}+0.3652C^{2}+0.094D^{2}$				
$\mathrm{DPPH}\;\left(\mu \mathrm{mol}\;\mathrm{AA}\;\mathrm{eq}/g\;\mathrm{dry}\;\mathrm{sample}\right)\;=92.82‐0.6692A‐0.415B+0.5242C+0.4025\mathrm{AC}‐0.49\mathrm{AD}‐0.565\mathrm{BD}+0.815\mathrm{CD}‐0.9563C^{2}‐0.4526D^{2}$				

*, ** and *** denote significance at *p* < .05, *p* < .01 and *p* < .001 respectively while ^NS^denotes not significant.

### 3.1 Effect of ultrasonic parameters on CA yield of ultrasound‐treated HS extract

The predictive equation obtained from the results for explaining the effectiveness of the ultrasonication pretreatments of HS extracts for optimal extraction of CA with high yield was written in coded terms after exclusion of insignificant variables as:

(5) $$\mathrm{CA}\;\mathrm{yield}\;\left(\mathrm{mg}/g\right)=2.44+0.3875A‐0.1917C‐0.1158D‐0.185\mathrm{AB}+0.78\mathrm{AC}+0.1225\mathrm{AD}+0.21\mathrm{BC}+1.7\mathrm{BD}‐0.24\mathrm{CD}+1.1A^{2}+0.7003B^{2}+0.3652C^{2}+0.094D^{2}\;\;\;\;\;\;\;\;\;\;\;\;\;\;\;\;\;\;\;\;\;\;\;\;\;\;\;\;\;\;\;\;\;\;\;\;\;\;\;\;\;\;\;\;\;\;\;\;\;\;\;\;\;\;\;\;\;\;\;\;\;\;\;\;\;\;\;\;\;\;\;\;\;\;\;\;\;\;\;\;\;\;\;\;\;\;\;\;\;\;\;\;\;\;\;\;\;\;\;\;\;\;\;\;\;\;\;\;\;\;\;\;\;\;\;\;\;\;\;\;\;\;$$

Among the linear terms: frequency (A) and temperature (C) were extremely significant factors that influenced CA yield, followed by time (D) while the effect of power density (B) was insignificant though positive (Table 2). All the interaction and quadratic terms showed a very significant (*p* < .01) effect for CA yield. The results showed that the experimental CA yield obtained varied from 1.34 ± 0.00 to 4.09 ± 0.01 mg/g; the maximum achieved at A = 60 kHz, B = 40 W/L, C = 40℃, and D = 35 min, while the minimum was obtained at A = 40 kHz, B = 30 W/L, C = 40℃, and D = 35 min. This suggested that lower frequency and power density pretreatment (sonication) conditions may not be desirable for ultrasonic extraction of CA with high yield from HS variety.

**FIGURE 1:**
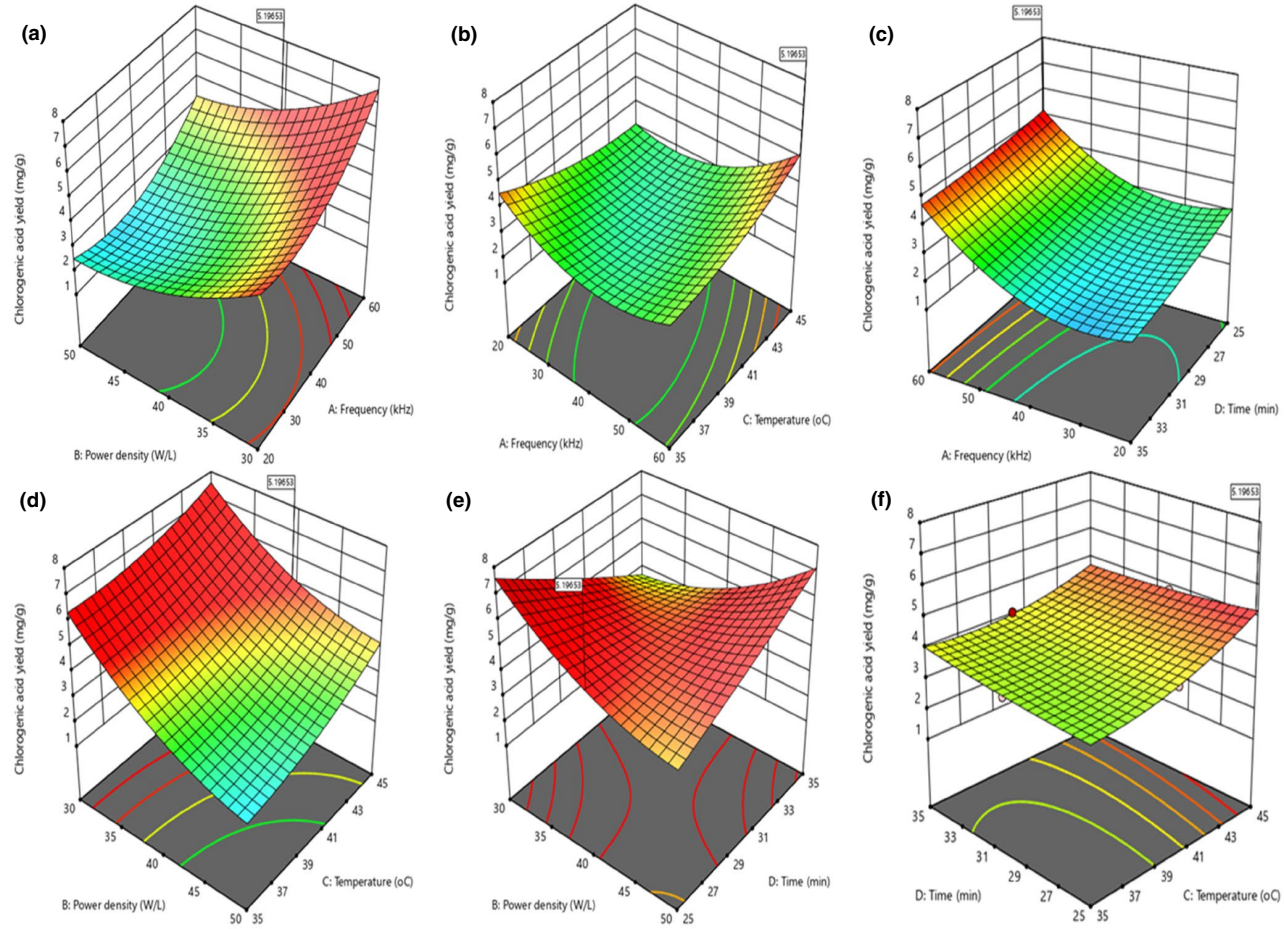
(a) Contour and response surface plots depicting the interactive influence of frequency, power density, temperature, and time on the CA yield of the UTHS sample

Figure 1 displayed the 3‐dimensional response surface (3‐D RS) and 2‐dimensional contour (2‐D C) plots of CA yield for UTHS sample as a function of the factors (A, B, C, and D). Representations of regression equation graphically (3‐D RS and 2‐D C plots) are very useful to evaluate independent‐dependent (factor‐response) variables relationship. Circular 2‐D C plot indicates insignificant interactions of parameters, while elliptical 2‐D C plot depicts significant interactions of variables (Cui et al., 2014). CA yield slightly decreased at first and then increased steadily as A increased and B decreased. Maximum CA yield was attained when A and B were 60 kHz and 30 W/L, respectively. Similarly, CA yield decreased slightly at first and then increased when both A and C increased, which was confirmed by the perturbation plot in Figure S1a. Figure 1; e revealed that BD interactions were significant owing to the elliptical 2‐D C plots and confirmed by the results in Table 2. CA yield increased with increased C and D. This finding was in similitude with the report of Banožić et al. (2019).

### 3.2 Effect of ultrasonic parameters on DPPH of UTHS extract

The DPPH model obtained through multiple regression analysis of the experimental data was reported in coded values after elimination of insignificant terms by the quadratic polynomial equation as:

(6) $$\mathrm{DPPH}\;\left(\mu \mathrm{mol}\;\mathrm{AA}\;\mathrm{eq}/g\;\mathrm{dry}\;\mathrm{sample}\right)\;=92.82‐0.6692A‐0.415B+0.5242C+0.4025\mathrm{AC}‐0.49\mathrm{AD}‐0.565\mathrm{BD}+0.815\mathrm{CD}‐0.9563C^{2}‐0.4526D^{2}\;\;\;\;\;\;\;\;\;\;\;\;\;\;\;\;\;\;\;\;\;\;\;\;\;\;\;\;\;\;\;\;\;\;\;\;\;\;\;\;\;\;\;\;\;\;\;\;\;\;\;\;\;\;\;\;\;\;\;\;\;\;\;\;\;\;\;\;\;\;\;\;\;\;$$

**FIGURE 2:**
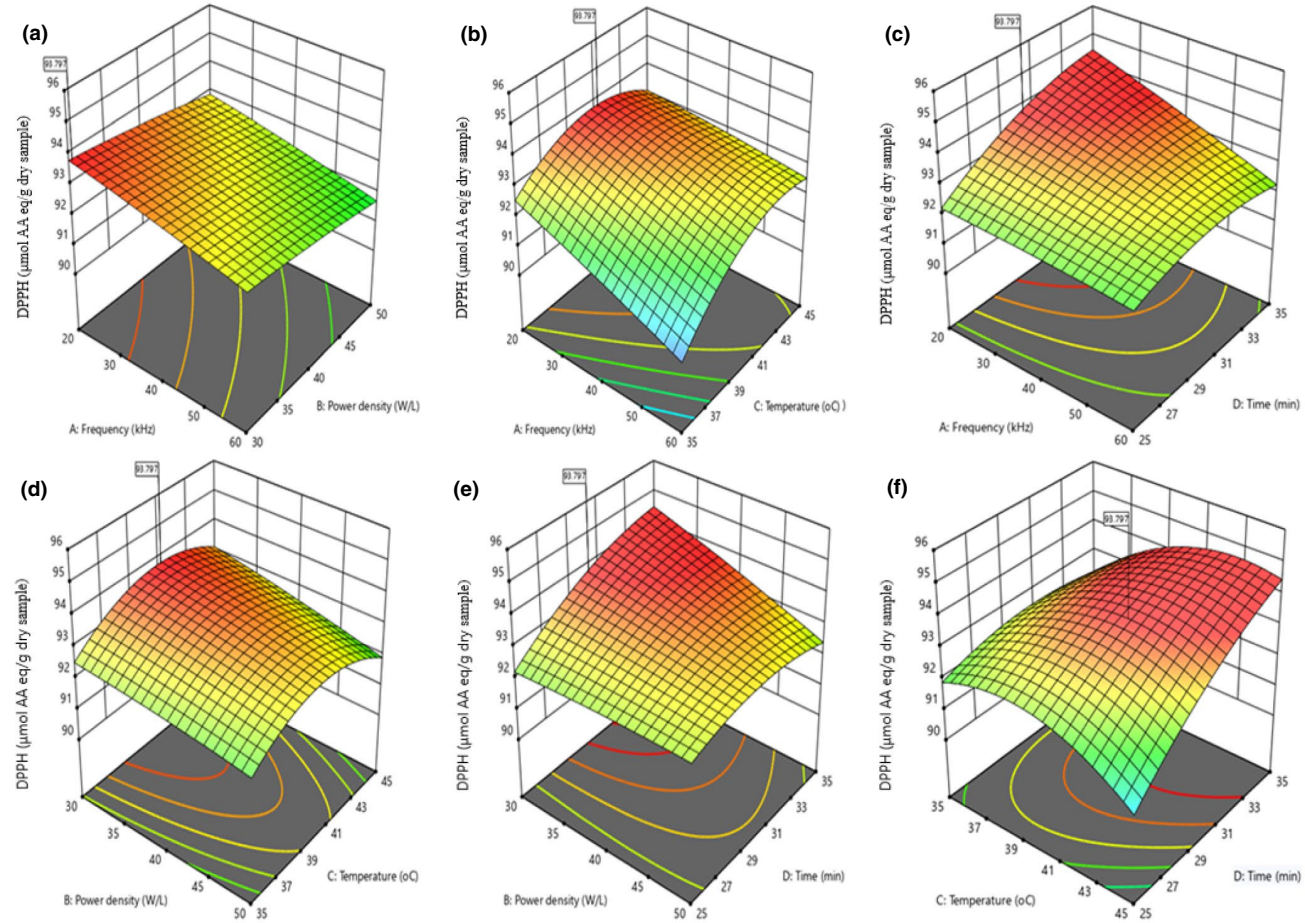
(b) Contour and response surface plots depicting the interactive influence of frequency, power density, temperature, and time on the DPPH of the UTHS sample

The linear terms A, B, and C significantly (*p* < .01) influenced the DPPH of the UTHS sample within a 99% confidence interval (Table 2). However, A was the extremely significant variable that influenced the DPPH of the UTHS sample. The results revealed that the interactions between A and C (AC), A and D (AD), B and D (BD), and C and D (CD) significantly influenced the DPPH of the UTHS sample. This was confirmed in Figure 2 by their elliptical 2‐D C plots. Consequently, as frequency decreased and temperature or time increased, the DPPH of the UTHS sample too increased (Figure 2; b and c). Statistically, the quadratic terms of A and B did not influence the DPPH of the UTHS sample. Figure 2; a–f showed that all the six 3‐D RS were convex in shape. This suggested that the ranges of the paramenters were correctly selected. Figure 2; a, b, d, and e showed that the DPPH of the UTHS sample steeply decreased when A and/or B increased, confirmed by the perturbation plot in Figure S1b. The results revealed that as temperature increased, the DPPH of the UTHS sample also increased gradually, first to somewhere above mid‐ranged temperatures (41–43℃) and then decreased from this point to 45℃ (Figure 2; d and f). From Figure 2; b, c, and f, the DPPH increased slowly with increased D to a maximum at 35 min. A similar result was reported by Mintah et al. (2019).

### 3.3 Model validation and verification

The reliability of the model equations obtained for prediction of the optimal response (CA yield and DPPH) values (4.990 ± 0.079 mg/g and 93.171 ± 0.369 μmol AA eq/g dry sample respectively) was tested under the obtained optimum conditions: A = 20.000 kHz, B = 30.000 W/L, C = 37.935℃, and D = 27.978 min. The optimal conditions were however modified, taken into consideration the real extraction operability as A = 20.0 kHz, B = 30.0 W/L, C = 37.9℃, and D = 28.0 min, under which the experimental DPPH and CA yield performed in triplicate were 93.197 ± 0.213 μmol AA eq/g dry sample, and 5.007 ± 0.033 mg/g, respectively. The relative error of the predicted and experimental value of CA yield was 0.0034 (0.34%) and that of DPPH was 0.0002 (0.02%) (Table S1b), compared very well with the predicted values and <5% (Boateng et al., 2020). This showed that the model was feasible and reliable hence acceptable for high bioactive CA extraction from HS variety.

### 3.4 Comparison of CA yield and DPPH from ultrasonic‐assisted extraction and extraction without ultrasonic treatment

**FIGURE 3:**
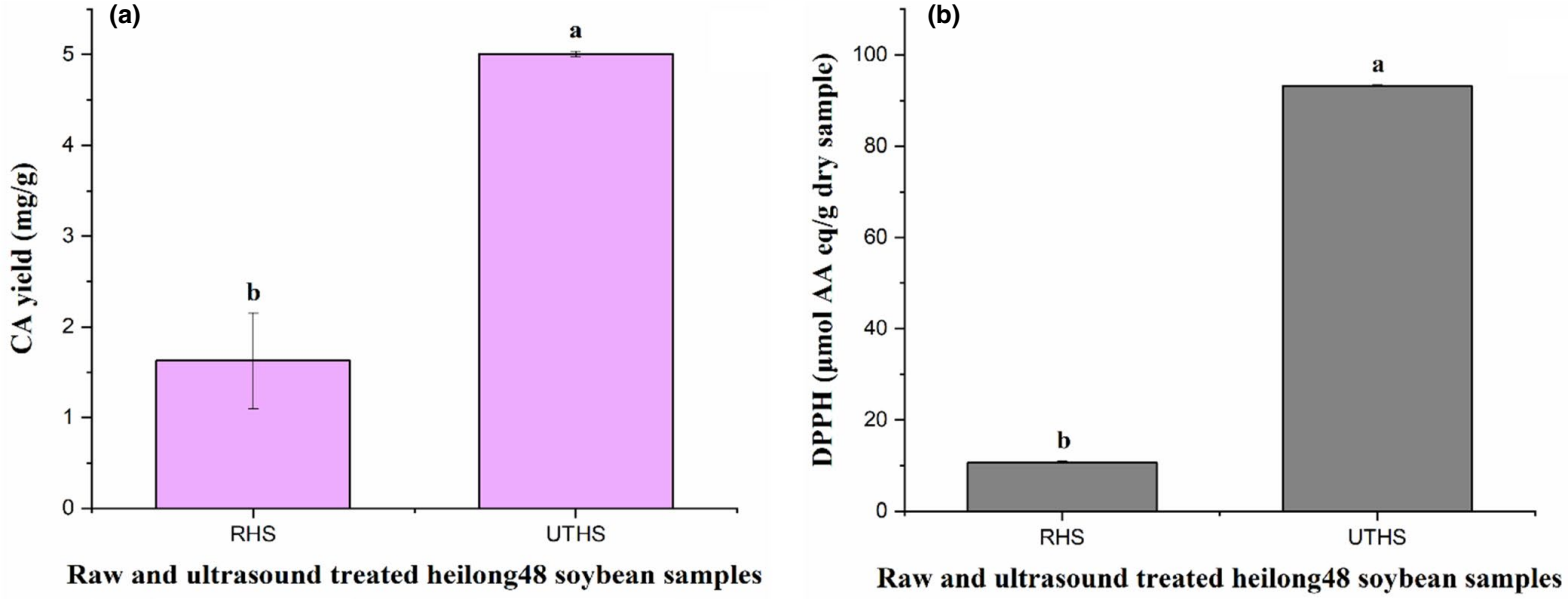
CA yield (a) and DPPH (b) of raw HS (RHS) and UTHS samples

DPPH (93.197 ± 0.213 μmol AA eq/g dry sample) and CA yield (5.007 ± 0.033 mg/g) obtained under the optimized ultrasonic‐assisted extraction conditions (frequency = 20.0 kHz, power density = 30.0 W/L, temperature = 37.9℃, and time = 28.0 min) were compared with that (DPPH, 10.760 ± 207 μmol AA eq/g dry sample and CA yield, 1.627 ± 0.528 mg/g) obtained without ultrasonic treatment as depicted in Figure 3. The results indicated that the ultrasonic‐assisted extraction gave higher CA yield with improved DPPH than extraction without ultrasound treatment. This was in accordance with the report of Mintah et al. (2019). Ultrasonication involves back and forth oscillation and collapsing of cavitation bubbles leading to shear thrusts, microjets, shock waves, and turbulence. Intense circulatory motion of the cavitation bubbles leads to microstreaming causing cell wall (of food samples) disruption, food matrix permeability, and mass transfer (Mintah et al., 2019). This implied that the cellular matrix of the UTHS sample was more permeable after the ultrasonic treatment and allowed the extraction solvent into its internal structure, which caused more CA to dissolve in solution, facilitating the extraction of CA with high yield and enhanced antioxidative activity from the UTHS sample.

### 3.5 Effect of UAE on the structure of UTHS sample

**FIGURE 4:**
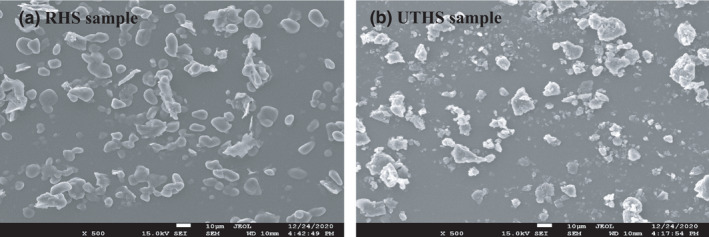
SEM micrograph of RHS and UTHS samples

Figure 4 depicted the microstruture of the HS samples: nonultrasound‐treated (raw HS; RHS) and ultrasound treated at obtained optimized conditions (UTHS). The particles of RHS sample were characteristically compact and smooth‐surfaced. The particles of the UTHS sample were, however, rough‐surfaced, porous, and loose. This indicated that the cavitation influence of the optimized ultrasonic conditions caused a sponge influence hence resulting in the loose structure of the UTHS sample. The cavitation also broke the bonds (covalent, noncovalent, hydrogen, and disulfide) between the molecules of the cell wall of the UTHS sample, which increased free CA mass flow. This explained the higher CA yield with the improved antioxidative activity obtained from the UTHS sample. These results were in similitude to that reported by Mintah et al. (2019).

### 3.6 Proximate

The proximate composition of the HS variety consisted of 42.583 ± 1.010% crude protein, 18.733 ± 0.116% crude fat, 4.587 ± 0.012% crude ash, 1.815 ± 0.005% crude fiber, and 7.650 ± 0.150% moisture (Table 3). These components give interconnected information about food quality (Desseva et al., 2020). The fat content was in similitude with that reported by Dixit et al. (2011). Likewise, the protein content agreed with that reported by Grieshop and Fahey (2001). The HS variety showed high protein and fat content. Proteins execute a prime function in the growth and maintenance of the human body and carbohydrates and lipids are the energy‐giving nutrients in food (Twinomuhwezi et al., 2020). As a result, these high protein and fat contents of HS variety obtained showed that it was a rich dietary protein (Dixit et al., 2011) and food energy source for human nutrition.

**TABLE 3:** Physicochemical properties of HS variety

Parameter	Mean ± SD (%)
Moisture content	7.650 ± 0.150^d^
Total solids	92.351 ± 0.150^a^
Crude protein	42.583 ± 1.010^e^
Crude ash	4.587 ± 0.012^b^
Crude fat	18.733 ± 0.116^c^
Crude fiber	1.815 ± 0.005^f^

Note

Superscripts that do not share the same letter are significantly different at *p* < .05.

The moisture content and crude ash were comparable to that reported by Etiosa et al. (2018). The water/moisture content and/or total solids of food are significant not only to give a basis for expressing on a dry or wet basis, the content of the other constituents but a significant factor in the quality and stability of food (Twinomuhwezi et al., 2020). The low moisture content implied that under good storage conditions, HS variety could have extended shelf life. HS variety had a high total solids content of 92.351 ± 0.150%. High total solids increase the nutritive value of products and improve the keeping quality (Odu et al., 2012). This suggested that the HS variety had a high nutritive value with improved keeping quality. The crude ash content suggested that the HS variety was a good source of valuable minerals. The crude fiber of HS variety obtained was lower than that (6.732 ± 0.013% and 7.001 ± 0.668% for Clark 63K and SCS 1 soybean varieties, respectively) reported by Muhsin and Zegeye (2018).

The proximate composition of the HS variety obtained showed that its quality was high. Nevertheless, food components are interrelated. Consequently, the proximate components of the HS variety are interrelated with its bioactive components. The most studied interaction is the protein–polyphenol interaction (Sęczyk et al., 2019). There is also protein–CA interaction reported by Wildermuth et al. (2016) where low molecular weight polypeptides are bound to soluble CA (about half). This suggested that some of the CA in the HS bonded to its proteins. Thus, resulted in a decreased quantity of free CA in the HS and would reduce the yield of CA extraction. The implication of this is that, when the protein content of HS increases, the yield of CA extraction will decrease.

### 3.7 Bioactive compounds

The TPC of the HS variety was 4.726 ± 0.002 mg GAE/g (Table 4). These results were higher than the result of Malenčić et al. (2008) and implied that HS variety had high TPC. However, the TFC of the HS variety was 0.040 ± 0.008 mg RE/g. Malenčić et al. (2012) reported a similar result. According to Maksimovíc et al. (2005), soybean hybrids are poor in flavonoids relative to some other industrial crops, like maize hybrids. This probably explained the low TFC obtained in this research. The TPC of the HS variety was significantly higher than TFC. A similar trend was reported by Prvulović et al. (2016). According to Aberoumand and Deokule (2008), plant polyphenols through the hydrogen‐donating property of their hydroxyl groups acts as reducing agents and antioxidants. This suggested that HS variety was a good source of plant antioxidants.

**TABLE 4:** One‐way ANOVA of bioactive compounds and antioxidant activity of HS variety

Parameter	Mean ± SD
Total flavonoids content (mg RE/g)	0.040 ± 0.008^f^
Total polyphenol content (mg GAE/g)	4.726 ± 0.002^c^
Total phenolic acids (mg GAE/g)	1.883 ± 0.005^d^
Chlorogenic acid content (mg/g)	1.627 ± 0.528^e^
DPPH (μmol AA eq/g dry sample)	10.760 ± 0.207^b^
FRAP (mg FeSO_4_/mg dry weight)	12.563 ± 0.280^a^

Note

Superscripts that do not share the same letter are significantly different at *p* < .05.

Martins et al. (2011) stated that more than half of the 8000 phenolic compounds that occur naturally, flavonoids constitute the major component. Nevertheless, this research had opposing results to our report, implying that the HS variety was richer in phenolic acids (with a CA content of 1.627 ± 0.528 mg/g) than flavonoids. The CA content of the HS obtained was lower than 62.10 ± 0.13 mg/g reported by Adane et al. (2019) in the bean and leaves of *Coffea arabica*. The TPA of the HS variety was 1.883 ± 0.005 mg GAE/g (Table 4). Our result was higher than 32 µg/g reported by Perumalla and Nayeem (2012). This implied that the HS variety was a suitable raw material for the extraction of CA. The result, therefore, strongly suggested that polyphenol content of HS variety was high and should be considered as an important raw material for CA extraction, as its pharmacologic influences (diuretic, anti‐inflammatory, or antioxidative activity) could be ascribed to the existence of these worthful components.

### 3.8 Antioxidant activity of heilong48 soybean variety

Antioxidative activity of soybean foods and seeds correlates with isoflavones and total phenolics positively (Devi et al., 2009). The antioxidant activity of the HS variety was shown in Table 4. This study employed DPPH and FRAP in the analysis of the antioxidant activity of HS variety, justifiable by the reason that one assay is inadequate to measure antioxidant activity reported by researchers. Xiao et al. (2014) reported that the single antioxidant property model hardly reflects the antioxidant capacity of samples because of the differences in the theoretical bases of different antioxidant measurements. A single assay, therefore, could be inadequate. The antioxidant activity of HS variety was 10.760 ± 0.207 μmol AA eq/g dry sample (measured by DPPH assay) and 12.563 ± 0.280 mg FeSO_4_/mg of dry weight (measured by FRAP assay). Since soybean comprises of different types of phenolic compounds, there is the possibility that their content and composition will influence its antioxidative activity level. There was a significant difference between DPPH and FRAP (*p* < .05). This trend was reported by Prvulović et al. (2016). The high antioxidant activity exhibited by HS variety suggested that it is a rich source of easily accessible plant‐based natural antioxidants that could be utilized as a potential food supplement or in the pharmaceutical industry.

### 3.9 Correlation between TPC, TPA, CA, TFC, DPPH, and FRAP

**FIGURE 5:**
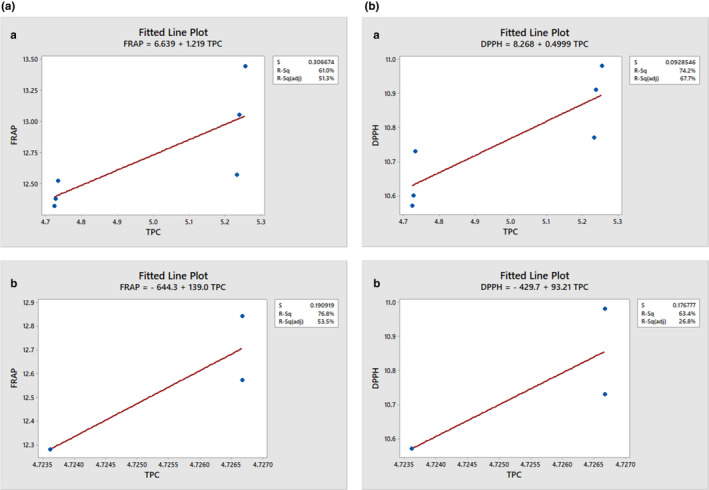
(a) Correlation [(a) *r* ^2^ = .610; (b) *r* ^2^ = .768] between TPC and FRAP of HS variety. (b) Correlation [(a) *r* ^2^ = .742, (b) *r* ^2^ = .634] between TPC and DPPH of HS variety

Higher TPC in the HS variety gave a maximal antioxidative activity, shown by a strong TPC and antioxidative activity correlation. Overall, FRAP had a stronger correlation with TPC than that between DPPH free radical scavenging activity and TPC (Table S2a). The TPC was highly correlated with scavenging ability against DPPH radical (*r* = .796) and FRAP (*r* = .876). Antioxidative activity increased proportionally to the TPC (Figure 5a; a and b; b; a and b). A linear correlation (*p* < .05) was established between DPPH‐radical scavenging activity and TPC with *r* ^2^ = .634 when three data points were used but had *r* ^2^ = .742 when six data points were plotted. This result agreed with that of Malenčić et al. (2008) where they obtained *r* = .66963 (in seed extracts of different soybean hybrids) for multiple data points plotted. Similarly, FRAP had a linear relationship (*p* < .05) with TPC but with a higher *r* ^2^ value (*r* ^2^ = .768) relative to that of DPPH (Figure 5a,b) when three data points were used. However, a lower *r* ^2^ value (*r* ^2^ = .610) than that of DPPH was obtained using six data points.

The higher FRAP and DPPH‐radical scavenging ability of HS variety might be attributed to the presence of higher phenolic contents of the studied sample, and this was in similitude with the report of Xiao et al. (2014). This relationship between the antioxidative activity and TPC of the HS variety indicated that phenolic compounds contributed about 74% (by DPPH) or 61% (by FRAP) of the antioxidant activity of the HS variety. Generally, less than five data points are too small in number to measure a strong correlation. Five or more data points are better and establish a stronger and significant correlation. This indicated that DPPH was a better parameter for measuring the antioxidant activity of HS variety than FRAP. Hence, even though both DPPH and FRAP had a significant positive correlation with TPC, DPPH had a stronger correlation with TPC than that of FRAP.

Both FRAP and DPPH activity is a measure of nonenzymatic antioxidative activity (Haida & Hakiman, 2019). As a result, the linear relationship obtained between FRAP and TPC showed that the HS variety had high antioxidant activity. According to Nam et al. (2014), the antioxidant activity of soybeans varies consistent with their genotype or ecological condition. Hence, the difference in our findings reference to that of Nam et al. (2014) might be due to this reason. However, the strong correlations between antioxidant activity and TPC of the HS variety supported the overall opinion that phenolic compounds are the most significant antioxidants of plant materials. DPPH assay is the most commonly used method for antioxidant capacity measurement. Its method differs in terms of kinetics and experimental conditions from that of FRAP. According to Ozgen et al. (2006), the DPPH method is more suited for samples with lipophilic antioxidants or those having a high lipid content. This might be the reason for the observed trend of correlation obtained between DPPH and TPC, and FRAP and TPC.

### 3.10 Protein‐fat‐polyphenol interaction

Phenolic compounds can have harmful nutritional influences by bonding with carbohydrates, lipids, and proteins (Alu'datt et al., 2013). Earlier studies by Bravo et al. (1992) reported interactions between plants’ phenolic compounds with other components (lipids, carbohydrates, and proteins) to occur frequently. Research shows that the chemical formation of phenolic compounds makes it possible for interaction between them and other food components via hydrophobic interactions, ionic bonding, hydrogen bonding, and covalent bonding (Alu'datt et al., 2019). Equation (7) showing the interaction between the protein, fat, and TPC of HS variety was assessed by multiple regression (*r* ^2^ = 1.000). Figures S2a,b showed the individual correlation between protein and TPC, and fat and TPC respectively.

(7) $$\mathrm{TPC}=4.800‐\left[0.001747\;\mathrm{Protein}\;\mathrm{content}\right]‐\left[0.000000\;\mathrm{Fat}\;\mathrm{content}\right]\;\;\;\;\;\;\;\;\;\;\;\;\;\;\;\;\;\;\;\;\;\;\;\;\;\;\;\;\;\;\;\;\;\;\;\;$$

The results showed that both protein and fat negatively correlated with total polyphenol, implying that an increase in protein and fat content results in a decrease in TPC. Protein, however, had a stronger negative correlation with TPC (*r* ^2^ = 1.000) than that between fat and TPC (*r* ^2^ = .246). This was also shown in the magnitude of their regression coefficients in the multiple regression equation (Equation 7). This result conformed to the finding of Alu’datt et al. (2013). Hence, there was an interaction between the protein and TPC of HS variety, limiting the quantity measured. This possibly indicated that the actual TPC of the HS variety was higher than that obtained. As a result, suggests the need to isolate protein from the bean before polyphenol, or CA extraction.

### 3.11 Influence of protein and fat on TPC and antioxidant activity of HS variety

**FIGURE 6:**
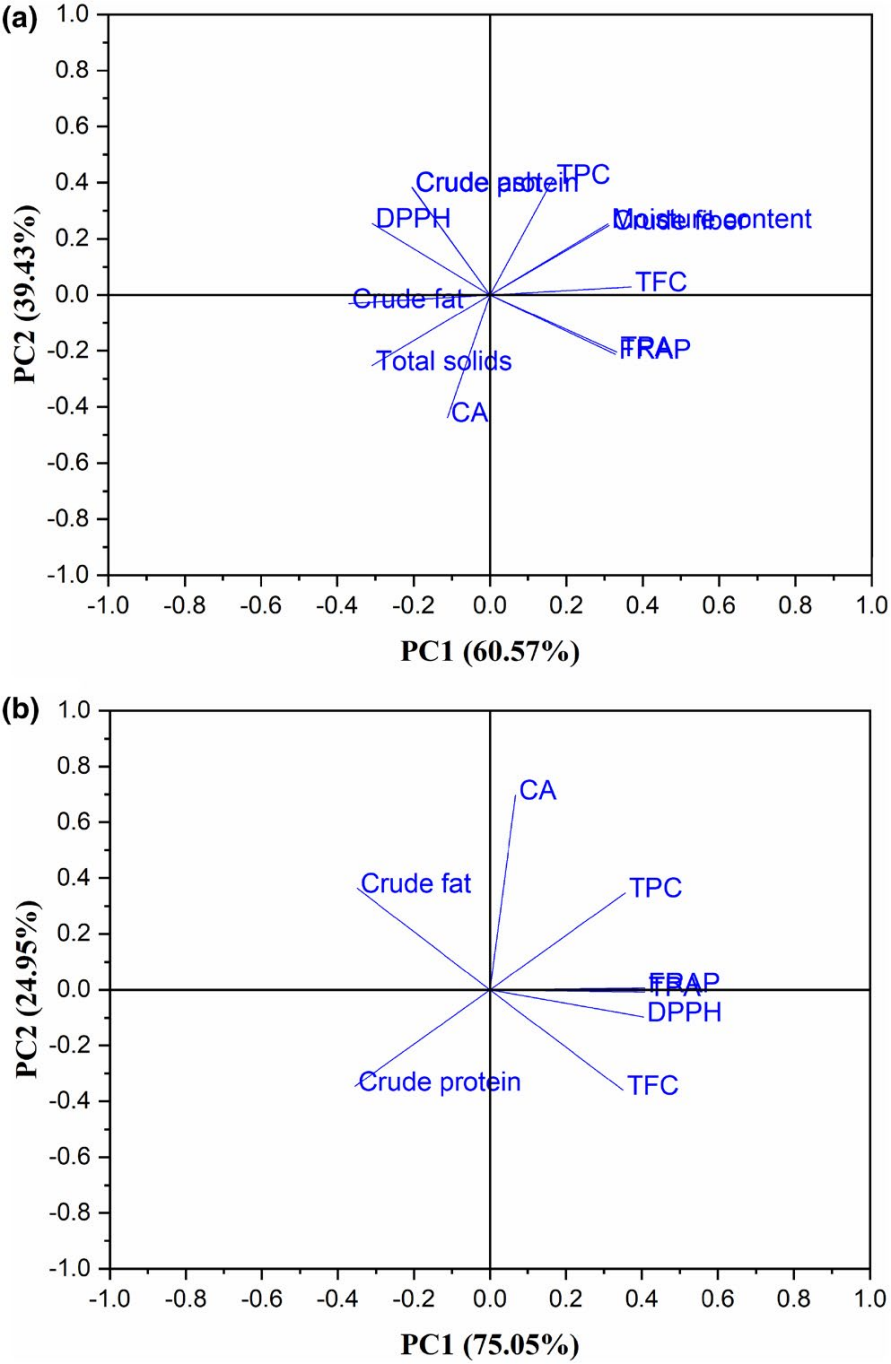
PCA: (a) Loading plot of moisture content, total solids, crude protein, ash, crude fat, crude fiber, TPC, TPA, CA, TFC, DPPH, and FRAP of HS variety. (b) Loading plot of crude protein, crude fat, TPC, TPA, CA, TFC, DPPH, and FRAP of HS variety

Principal component analysis displays similarities and differences among variables from their spatial distribution. PCA is statistically a useful technique. According to Mishra et al. (2013), it is applied in the reduction of the original variables (moisture content, total solids, fat, protein, crude ash, crude fiber, TPC, TPA, CA, TFC, DPPH, and FRAP) in a lesser numeral of underlying parameters (principal component) to divulge the interrelations between the different parameters and find the best numeral of extracted principal components (PC). Protein and fat values were considered as independent variables, while TPC, their phenolic acids (CA), DPPH, and FRAP were the dependent variables. Parameters were centered on two main PC, PC1 that explained 60.573% of the variability, and PC2 that explained 39.430% among the data (Figure 6a). Thus, both PC1 and PC2 explained 100.000% of the total data variance in the data set of the measured variables of HS variety. Figure 6b had a similar result.

PC1 elucidated the original variance's prime parts and PC2 consecutively explicated the original variance's minor parts. This suggests that the same PC described correlated parameters and dissimilar PC described less correlated parameters (Mishra et al., 2013). From Figure 6a,b, PCA revealed that all the studied, measured parameters were clustered into four quadrants. The loading plot's right side was occupied by most of the biochemical variables and amid these variables, the TPC was the right topmost parameter for both PC1 and PC2 (Figure 6a). While, half of the proximate parameters were on the left side of the loading plot (Figure 6a). On the contrary, Figure 6b had all the phenolic compounds and antioxidant activity on the right side of the loading plot, and only crude fat and protein were on the left side of the loading plot. CA recorded the highest positive loading for both PC1 and PC2 on the right upper side of the loading plot (Figure 6b).

From Figure 6a, the first quadrant consisting of protein, TPC, moisture content, crude fiber, and TPC exhibited a strong correlation with both PC1 and PC2 positively, while the second quadrant containing FRAP and TPA correlated with PC1 positively and with PC2 negatively. The third quadrant, which correlated with PC1 and PC2 negatively, had CA, total solids, and crude fat. The fourth quadrant, however, contained DPPH and crude ash indicated high positive loading with PC2 and negative to PC1. The results revealed too that HS variety contained higher TPC and DPPH scavenging activity. In Figure 6b, CA, TPC, TPA, and FRAP constituted quadrant one, DPPH and TFC made up quadrant two, only crude protein and fat were in quadrants three and four respectively. CA, TPC, TPA, and FRAP located in the same quadrant, indicated no existence of differences between the antioxidant mechanisms of these compounds. This interpretation suggested that the HS variety could be considered for its higher TPC, CA, and antioxidant parameters, and possibly be used for the extraction of CA. A strong negative correlation between TPC and crude protein proposed that there was an interaction between them. This implied that TPC decreases with increasing crude protein content and vice versa through bonding as stated by Alu’datt et al. (2013). When the protein content of HS is high, more of its polyphenols will be bonded to it. This reduces the quantity of free polyphenols in the HS and subsequently decreases the amount of CA in it. Pearson's correlation analysis indicated a similar result (Table S2b).

## 4 CONCLUSION

In the current research, ultrasonic parameters for bioactive CA extraction were optimized to achieve high CA yield coupled with the evaluation of the physicochemical and bioactive properties of HS variety. The study established an acceptable model for CA extraction with high yield and increased antioxidative activity from soybean (HS variety) with a desirability function of 0.918. SEM results confirmed the structural alterations of HS variety caused by the optimized ultrasonic parameters. Also, HS variety significantly contained high TPC, TPA, and CA as well as high antioxidant properties. A stronger FRAP relative to DPPH free radical scavenging activity was established. And protein–phenolic interaction (negative correlation—high protein content, results in low TPC) in the HS variety was realized. The studied sample had low moisture content and so could have extended shelf life under good storage conditions. It contained high ash content signifying a good source of minerals and relatively low fat. The use of HS variety for CA extraction could be economical since it is cheap and available all year round. Further studies should focus on the extraction of CA from this soybean (HS variety) using solid‐state fermentation (SSF), by examining the SSF parameters for maximum yield.

## CONFLICT OF INTEREST

The authors declare that they do not have any conflict of interest.

## AUTHOR CONTRIBUTIONS

**Nelson Dzidzorgbe Kwaku Akpabli‐Tsigbe:** Conceptualization (equal); Data curation (equal); Formal analysis (equal); Investigation (equal); Methodology (equal); Software (equal); Validation (equal); Visualization (equal); Writing‐original draft (equal); Writing‐review & editing (equal). **Yongkun Ma:** Funding acquisition (equal); Project administration (equal); Resources (equal); Supervision (equal). **John‐Nelson Ekumah:** Investigation (equal). **Juliet Osabutey:** Writing‐review & editing (equal). **Jie Hu:** Investigation (equal). **Manqing Xu:** Investigation (equal). **Nana Adwoa Nkuma Johnson:** Investigation (equal).

## ETHICAL APPROVAL

This study does not involve any human or animal testing.

## Supporting information

Supplementary Material

Supplementary Material

## Data Availability

The data that support the findings of this study are available from the corresponding author upon reasonable request.
