# Supplementary material for: Single‐frequency ultrasonic extraction of bioactive chlorogenic acid from heilong48 soybean variety: Parametric optimization and comprehensive evaluation of physicochemical and bioactive properties
Source: Food Sci Nutr. 2021 Oct 31;10(2):374–87. doi: 10.1002/fsn3.2593 (PMC8825731; doi:10.1002/fsn3.2593)
Supplement: Supplementary file 2 — Supplementary Material [file FSN3-10-374-s001.docx]

**Supporting information 2**

**Single-frequency ultrasonic extraction of bioactive chlorogenic acid from heilong48 soybean variety: parametric optimization and comprehensive evaluation of physicochemical and bioactive properties**

**Nelson Dzidzorgbe Kwaku Akpabli-Tsigbe^a,b^, Yongkun Ma^a*^,** **John-Nelson Ekumah^a,b^, Juliet Osabutey^c,d^, Jie Hu^a^, Manqing Xu^a^, Nana Adwoa Nkuma Johnson^a^**

^a^School of Food and Biological Engineering, Oversea College of Education, Jiangsu University, 301#, Xuefu Road, Zhenjiang 212013, Jiangsu, P. R. of China.

^b^Department of Nutrition and Food Science, College of Basic and Applied Sciences, University of Ghana, P. O. Box LG 134, Legon-Ghana.

^c^Department of Early Childhood Education, University of Education, P. O. Box 25, Winneba, Ghana.

^d^Virtuous Experimental School, P. O. Box AH 106, Achimota-Accra, Ghana, West Africa.

*Corresponding author: Email: [mayongkun@ujs.edu.cn](mailto:mayongkun@ujs.edu.cn) (Yongkun Ma)

**FIGURE CAPTIONS**

Figure S1. Perturbation plot for (a) CA yield (b) DPPH of ultrasound treated HS sample

Figure S2. Protein-fat-polyphenol interaction: (a) Correlation (*r^2^* = 1.000) between total polyphenol content (TPC) and crude protein content of heinong48 soybean variety. (b) Correlation *(r^2^* = 0.246) between TPC and crude fat content of heinong48 soybean variety.

| 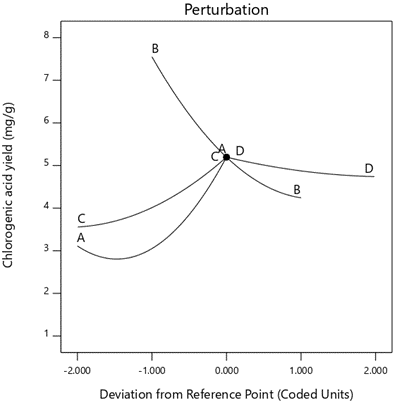  **(a)**  **(b)** | 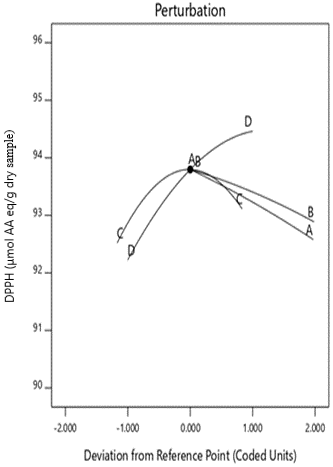 |
| --- | --- |

**Figure S1. Perturbation plot for (a) CA yield (b) DPPH of ultrasound treated HS sample**


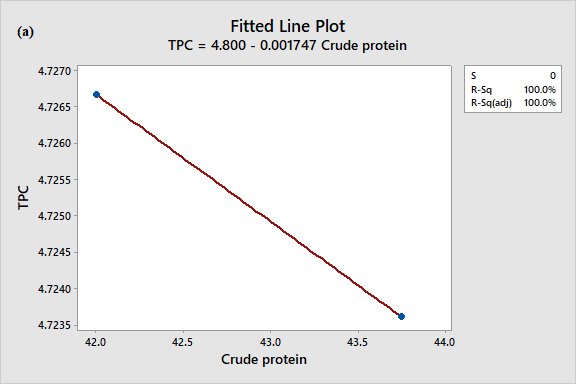


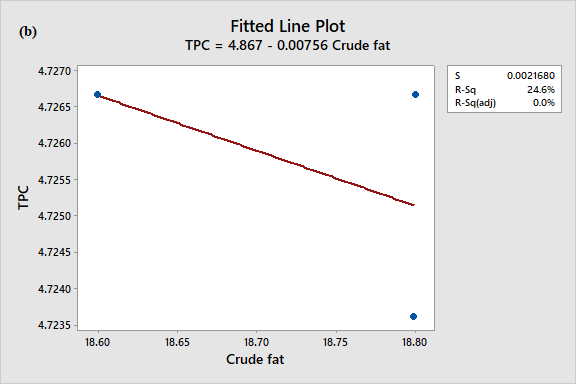


**Figure S2. Protein-fat-polyphenol interaction: (a) Correlation (*r^2^* = 1.000) between total polyphenol content (TPC) and crude protein content of heinong48 soybean variety. (b) Correlation (*r^2^* = 0.246) between TPC and crude fat content of heinong48 soybean variety.**
